# Fine and coarse roots of *Pinus ponderosa* trees: contributions to belowground carbon and nitrogen pools

**DOI:** 10.3389/fpls.2026.1969066

**Published:** 2026-10-21

**Authors:** Antonio Montagnoli, Mattia Terzaghi, Bruno Lasserre, Donato Chiatante, R. Kasten Dumroese

**Affiliations:** 1 Department of Biotechnology and Life Science, University of Insubria, Varese, Italy; 2 Department of Biosciences, Biotechnology and Environment, University of Bari Aldo Moro, Bari, Italy; 3 Department of Biosciences and Territory, University of Molise, Pesche, Italy; 4 United States Department of Agriculture, Forest Service, Rocky Mountain Research Station, Moscow, ID, United States

**Keywords:** carbon pool, forest vegetation simulator, modeling, nitrogen pool, ponderosa pine, root system

## Abstract

The fine, coarse, and tap roots of 32-year-old *Pinus ponderosa* growing at 1075 trees ha^-1^ in the Rocky Mountains of Idaho, USA, had biomass of 4.6, 42.2, and 27.8 Mg ha^-1^, respectively, containing 34 Mg carbon ha^-1^ and 125 kg nitrogen ha^-1^. Compared with these direct measurements, the base equations used in the United States Department of Agriculture Forest Vegetation Simulator use a higher carbon concentration (50%) but generate a lower total biomass (52.4 vs 74.7 Mg ha^-1^). Measuring root systems is complex and challenging, so this result is unsurprising given the few studies available for models that sampled belowground tree biomass compared to those for aboveground traits. Moreover, belowground root traits, including those of fine roots observed in this study, vary across a range of genetic, edaphic, and/or silvicultural factors. Thus, recent calls to improve forest carbon modeling requests that researchers prioritize increasing the sampling and reporting of belowground biomass. These data contribute to that effort.

## 1 Introduction

For forest trees, roots are the most undersampled component (Frank et al., 2019) because accessing the complex root system within the soil profile is inherently difficult (Nadelhoffer and Raich, 1992). Obtaining complete root systems of mature trees requires time-consuming, labor-intensive, destructive approaches. Root systems are usually excavated by using one or more of several principal methods, including, alone or in combination, hand shoveling, pneumatics, machine excavators, and explosives (e.g., Colombi et al., 2015; Chiatante et al., 2019; Zhang et al., 2021). Subsequently, for example, most biometric work on *Pinus ponderosa* Lawson & C. Lawson, a commercially important species abundant across 11 million ha in the western United States, has focused on aboveground characteristics, with only limited efforts to characterize the root systems of trees beyond the seedling stage (see discussions in Dumroese et al., 2019, and Zhang et al., 2021).

Unfortunately, the lack of robust data on root development and subsequent carbon (C) allocation has hindered the development of accurate models for assessing the amount of C added to the soil (Sinsabaugh et al., 1991). Holistic models of forest C dynamics that include above- and below-ground estimates provide insights into growth, biomass allocation, sequestration, soil health and productivity, and harvesting effects. These models are fundamental to the decision-making process of land managers and policymakers. Thus, a recent survey of ecosystem C modelers representing academia, government, industry, and non-profit sectors revealed that belowground carbon modeling was a high priority, as was the overall refinement of C models (Woodall et al., 2025); both priorities require additional, empirical belowground data.

In 2017, we initiated a root excavation study on 32-year-old *P. ponderosa* growing in the northern Rocky Mountains of the United States that were established from nursery-grown container seedlings. Our primary objective was to investigate potential long-term effects of chemical root pruning, but recognized this experiment could provide valuable, basic insights into coarse root development. Our *a priori* methods were focused, therefore, on examining overall coarse root architecture, ongoing root initiation and directional responses of roots to mechanical forces, and root system and individual root responses to nursery practice and slope (Dumroese et al., 2019, 2022; Montagnoli et al., 2019a, 2020). We also compared, *a posteriori*, our direct measures of coarse roots with those obtained using Terrestrial Laser Scanning (Montagnoli et al., 2024).

This study, conducted *a posteriori*, has the objective of adding empirical data to the relatively few publications concerning belowground C to help meet the expressed priority for more and refined belowground modeling of C. Therefore, we present new empirical measures of fine root (< 2 mm diameter) biomass and C and nitrogen (N) concentrations of the fine and coarse (≥ 2 cm) roots. We combine these data with previously reported data about coarse root biomass and above-ground metrics of these same trees (Dumroese et al., 2019; Montagnoli et al., 2021) to estimate C and N pools. We contrast our pool estimates with those obtained with equations that support the Forest Vegetation Simulator (FVS), the official national software suite used by the United States Department of Agriculture, Forest Service, which holistically models forest growth across a variety of anthropogenic and natural disturbance factors and their interactions.

## 2 Methods

### 2.1 Study site

Our 32-year-old *P. ponderosa* trees were growing at about 1000 m elevation on the University of Idaho Experimental Forest in northern Idaho, USA (lat 46.842240, long -116.871035; see map in Montagnoli et al., 2019a). Stand density was 1075 trees ha^-1^. This forest receives about 965 mm of annual precipitation (October through June with a summer drought) and the average annual air temperature is 7.2 °C with ~100 frost-free days (Soil Survey Staff, 2013). Prevailing winds are west-southwest during the growing season (Western Regional Climate Center, 2025). The habitat type is a *Thuja plicata*/*Clintonia uniflora*/*Clintonia uniflora* phase supporting mixed conifer species (Cooper et al., 1991). Trees were growing on a ~1.5-m deep Vassar series soil (Typic Udivitrands; Andisol) formed in volcanic ash above material weathered from granite (Soil Survey Staff, 2013; see Dumroese et al., 2019, for profile descriptions). The site, with a northeast aspect and slopes of 30 to 50%, was clearcut-harvested and broadcast-burned during 1985, with little reduction of the forest floor layer.

### 2.2 Tree selection and coarse root system excavation and measurement

Full descriptions of stand origin, early sampling, the tree selection process, root excavation methods, coarse root digitization, and coarse root trait analyses can be found in Dumroese et al. (2019) and Dumroese et al. (2022). To summarize, after the first growing season (1986), seedlings grew at 2-m x 2-m spacing without any treatment (e.g., irrigation, thinning, weed control). In 2017, we initially selected 15 trees and measured diameter breast height (DBH, cm) and fine roots (diameter < 2 mm); of those 15 trees, 10 were selected for felling and coarse root (including taproot) excavation (**Table 1**). For each felled tree, we used an air-spade to remove the soil from the coarse roots (diameter > 2 cm) to about 1.5 m from the trunk and to bedrock (about 1.5 m). Excavated coarse root systems were discretized, and we used those data, which are available in Montagnoli et al. (2021), to determine values for root traits (i.e., topology, diameter, and volume).

**Table 1:** Measured diameter breast height (DBH) and coarse (≥ 2 cm diameter) and tap root volume (cm^3^), and model estimates (Jenkins et al., 2003) for total aboveground biomass (TAB), the ratio of TAB to roots, and root biomass of ten, 32-y-old *Pinus ponderosa* trees.

	Measured		Modeled		
Tree ^a^	DBH (cm) ^a^	Coarse and tap root volume (cm^3^) ^a^	TAB (kg)	TAB:root	Root biomass (kg)
P1	24.1	86031	183.60	0.216	39.58
P4	24.1	70942	183.60	0.216	39.58
P5	25.6	91931	212.68	0.215	45.77
P6	23.3	54242	169.11	0.216	36.49
P8	34.2	207579	430.53	0.214	92.06
P9	27.8	94634	259.96	0.215	55.84
P11	27.1	98744	244.31	0.215	52.51
P12	21.4	32060	137.48	0.216	29.74
P13	28.6	122042	278.55	0.215	59.79
P15	23.1	46189	165.60	0.216	35.74
Avg	25.9	90439	226.54	0.215	48.71

^a^ Tree identification, DBH, and coarse root volumes per Montagnoli et al. (2021).

### 2.3 Fine root sampling

In early June, at a distance of 65–80 cm from the stem of each of the 15 trees, we collected four soil core samples corresponding to cardinal directions (North, downslope; South, upslope; East, leeward; West, windward). We carefully collected the forest floor within a 30-cm-diameter metal ring before using a custom, slide hammer soil coring device (8-cm diameter x 30-cm deep; Jurgensen et al., 1977) to obtain a sample. In the field, each core was subdivided into 10-cm samples (15 trees x 4 directions x 3 depths = 180 total). The forest floor and soil samples were placed into separate plastic bags and stored at 4 °C immediately after coring in a field-portable refrigerator until processing in the laboratory (≤ 10 days; walk-in cooler). Fine root traits were quantified for each forest floor and mineral soil sample. The washing, hand separation, stereomicroscopic examination, and scanning of fine roots were conducted in water to prevent desiccation and maintain root integrity. Each sample was manually washed over a 2-mm sieve, and fine roots (diameter < 2 mm) were hand-separated from soil, rocks, and debris, and examined under a stereomicroscope to segregate *P. ponderosa* roots from other plant species based on color, texture, and shape. When the root origin was in doubt, we used a transverse cut on fresh root material to identify the plant species’ fine roots. All fine root samples were scanned at a resolution of 800 dpi with a calibrated flatbed scanner coupled to a transparency unit for image acquisition (Epson Expression 10,000 XL) and analyzed with root scanning software (WinRhizo Pro V. 2007d; Regent Instruments Inc., Quebec, Canada). Samples were then oven-dried at 70 °C to constant mass and weighed to obtain biomass. We calculated specific root length (SRL) as the quotient of fine root length (m) and dry biomass (g).

### 2.4 Coarse roots wood sampling

We used a drill on each of the ten excavated trees to extract a wood sample from six different positions of the root system (**Figure 1**). Three samples were from the taproot (approximately top, middle, and bottom). The other three samples were from within the cage (i.e., the cylindrical region centered at the taproot composed of the zone of rapid taper of horizontal surface roots as well as the numerous sinker and deep roots that enmesh a large mass of soil; Danjon et al., 2005) (10 trees x 6 positions (3 taproot; 3 first order lateral) = 60 total). To include potential differences due to root eccentricity in the first-order lateral roots, we sampled proximal to the taproot, from the top of the root downward to the centroid (upper), and from the bottom of the root upward to the centroid (lower) (**Figure 1**). The first 5–10 mm of core wood shavings (mostly bark) were discarded when sampling.

**Figure 1:**
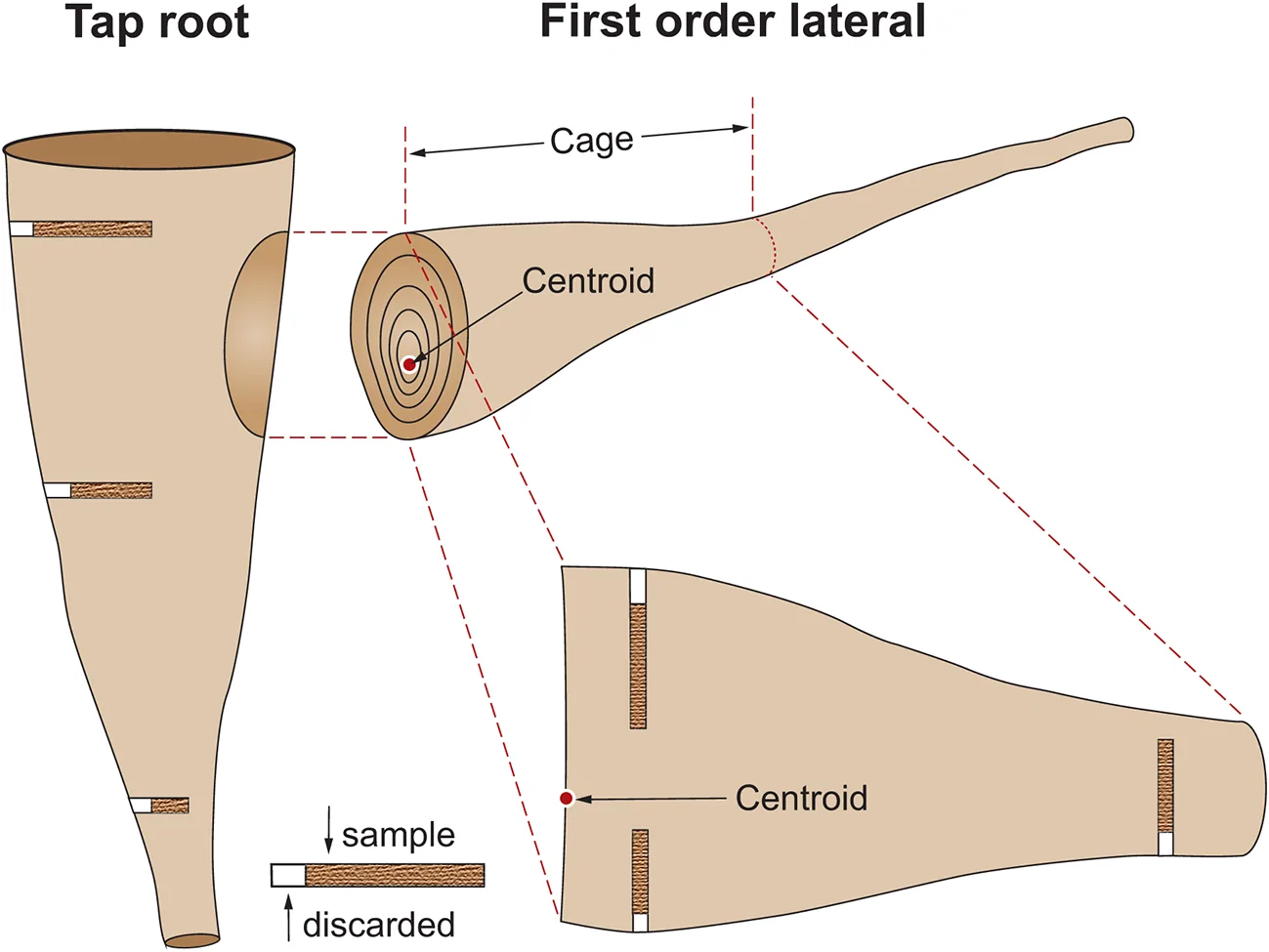
Sampling locations on the tap and first-order lateral roots of *Pinus ponderosa* for carbon and nitrogen analyses.

### 2.5 Wood density

We randomly selected ten lateral roots from across our suite of excavated coarse roots, systematically ranging in maximum diameter (from 3 to 15 cm), and cut a 1-cm-thick disk. Disks were oven-dried at 70 °C to constant mass, and weights were recorded. We measured perpendicular diameters to calculate cross sectional area. Disks were quickly immersed in melted paraffin wax, allowed to drain and cool, and reweighed (Blake and Hartge, 1986). The thinly coated disks were immersed in water to determine volume (ml^3^) displacement (Harrington et al., 1994). Density (kg m^-3^) equals the uncoated disk dry mass and volume quotient.

### 2.6 Carbon and nitrogen analyses

Fine roots from each tree and within a depth were pooled by cardinal direction (15 trees x 4 directions = 60 samples). Fine roots and the drill samples (Section 2.4) were dried at 70 °C to constant weight, coarse ground as necessary to pass a 2-mm sieve, and fine ground (homogenized) with a ball mill to pass a 0.44-mm mesh. Samples were analyzed for C and N concentration by dry combustion in a high-temperature induction furnace with C and N determinators (TruSpec CN Analyzer; LECO Corp., St. Joseph, Michigan, USA).

### 2.7 Root biomass and C and N pools

We used the soil core radius (4 cm) to determine the area (50.27 cm^2^) to scale the mean fine root biomass (trees x locations; n = 54) to a hectare. For coarse roots, we converted the volumes of each lateral root order and taproot to biomass (n = 10 for each root type) using the wood density value we determined for our range of root diameters. The biomass per tree was scaled to hectares using 1075 trees ha^-1^. Total C (Mg ha^-1^) and N (kg ha^-1^) in those tissues were the product of biomass by element concentration. Calculations were performed independently for each tree and soil depth for fine roots, and for each tree for coarse roots (laterals and taproots), before calculating means and standard errors. Thus, the variability associated with biomass and elemental concentrations was incorporated into the individual pool estimates.

### 2.8 FVS estimates of biomass and total C

As noted in the Forest Vegetation Simulator (FVS) Fire and Fuels Extension (Rebain, 2010), the model uses equations from Jenkins et al. (2003) to calculate tree biomass in the United States. We used the diameter at breast height (DBH; cm) values from our 10 trees to estimate total aboveground biomass (TAB; kg) using the equation presented in table 4 (pine) of Jenkins et al. (2003):


TAB = Exp(β0 + β1 ln(DBH)),

where DBH = cm, β0 = -2.5356, and β1 = 2.4349.

We also used our average DBH (cm) values to estimate the ratio of roots for each tree, using the equation presented in table 6 (softwoods) of Jenkins et al. (2003):


ratio = Exp(β0 + (β1/DBH)),

where β0 = -1.5619, and β1 = 0.6614.

Thus, root biomass was estimated as the product of TAB and ratio. We estimated the C pool using the Jenkins et al. (2003) biomass estimates and the default FVS C concentration (0.50), and compared both estimates with our direct measurements, assuming 1075 trees ha^-1^.

### 2.9 Statistical analyses

We examined data for normality and homogeneity of variance prior to analysis. When necessary, we transformed the data to meet the assumptions of parametric tests. Differences in fine root traits, C and N concentrations, and carbon-to-nitrogen ratios (C:N) among soil depths (forest floor and mineral soil) were assessed using linear mixed-effects models, with soil depth included as a fixed effect and tree identity as a random effect to account for the non-independence of samples collected from the same tree. When significant effects were detected, we used Bonferroni’s *post hoc* test to identify pairwise differences among depths. Statistical significance was accepted at p < 0.05. We used SPSS 25.0 (IBM Corp., Armonk, NY, USA).

## 3 Results

Sampling depth for fine roots was significant (all p <0.001) for length, diameter, SRL, and biomass (**Figure 2**; **Table 2**). For each variable, roots in the forest floor differed from those at all other depths in the mineral soil, and the latter were not significantly different. Compared to the fine roots in the different depths of mineral soil, fine roots in the forest floor had less total biomass (10.4 ± 1.8 vs. 142.2 ± 5.0 g m^-2^) and length (186 ± 30 vs. 1878 ± 77 m m^-2^), smaller diameter (0.44 ± 0.01 vs. 0.54 ± 0.01 mm), and higher SRL (21.2 ± 1.0 vs. 13.6 ± 0.3 m g^-1^) (**Figure 2**).

**Figure 2:**
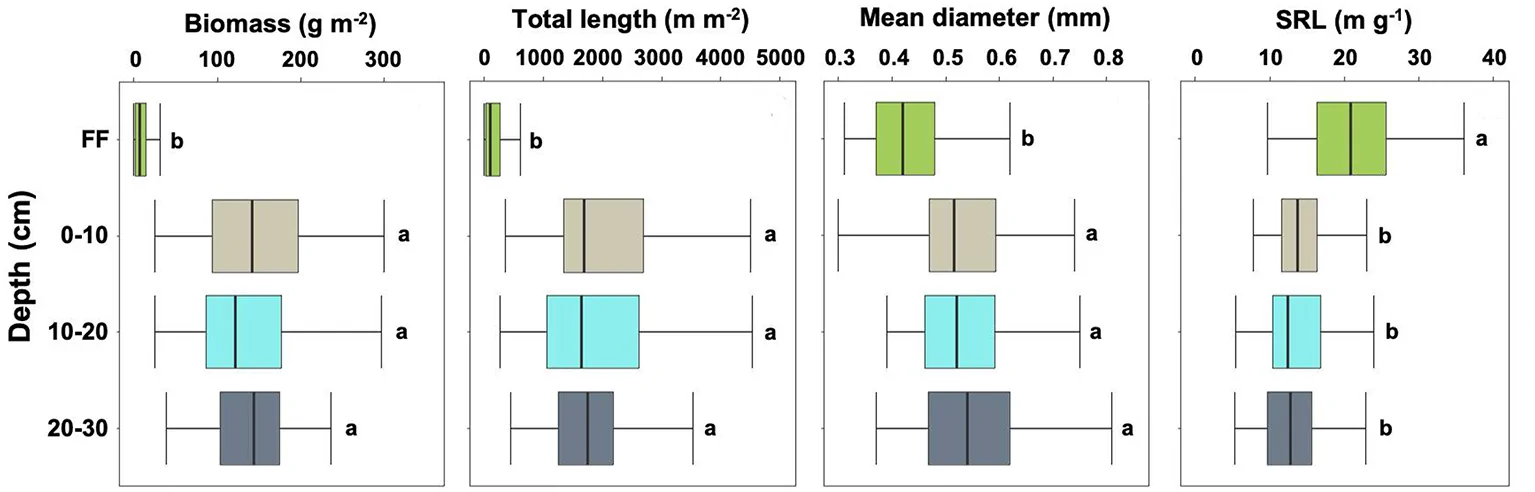
Distribution of biomass, total length, mean diameter, and specific root length (SRL) of fine roots (≤ 2 mm) in the forest floor (FF) and mineral soil at three 10-cm depth intervals. Values for n range from 44 to 53. Each box represents the interquartile range (25th–75th percentiles). Vertical lines indicate the median and whiskers extend to the most extreme values within 1.5 times the interquartile range. Different letters indicate significant differences among soil layers within each fine-root trait (Bonferroni test, p < 0.05).

**Table 2:** Mean values (± standard error) for carbon (C) and nitrogen (N) concentrations and C to N ratio (C:N), biomass, and estimated C (Mg ha^-1^) and N (kg ha^-1^) pool size for fine roots (≤ 2 mm) growing in the forest floor and in mineral soil at three depths, and for coarse lateral roots by order and the taproot.

Root type	Fine root depth and coarse root order	Root biomass	C (%) ^a^	N (%) ^a^	C:N ^a^	Biomass (Mg ha^-1^)	C (Mg ha^-1^)	N (kg ha^-1^)
Fine	Depth (cm)	Biomass (g m^-2^) ^b^						
	Forest floor	10.4 (1.8) a	41.0 (0.5)	1.05 (0.06) a	39.0 (2.3) a	0.10 (0.02)	0.04 (0.01)	1.09 (0.19)
	0-10	146.7 (8.9) b	41.2 (0.4)	0.93 (0.03) b	44.3 (1.4) ab	1.47 (0.09)	0.60 (0.04)	13.62 (0.83)
	10-20	138.1 (10.6) b	40.9 (0.3)	0.74 (0.04) c	58.5 (4.4) bc	1.38 (0.11)	0.56 (0.04)	10.21 (0.78)
	20-30	141.5 (6.7) b	42.0 (0.4)	0.65 (0.04) c	68.5 (5.5) c	1.41 (0.07)	0.59 (0.03)	9.26 (0.44)
	Total	464.3 (23.0)	–	–		4.64 (0.23)	1.80 (0.12)	34.18 (2.24)
Coarse	Order	Biomass (kg tree^-1^) ^c^						
	First	29.71 (5.64)	46.1 (0.1) ^d^	0.130 (0.004) ^d^	361.8 (15.0) ^d^	31.9 (6.1)	14.72 (2.88)	41.5 (7.9)
	Second	8.22 (1.24)	–	–	–	8.8 (1.3)	4.08 (0.62)	11.8 (1.8)
	Third	1.27 (0.34)	–	–	–	1.4 (0.4)	0.63 (0.2)	1.9 (0.5)
	Total lateral	39.29 (6.77)	–	–	–	42.2 (7.3)	19.4 (3.6)	55.1 (10.1)
	Taproot	25.84 (2.98)	45.9 (0.1) ^e^	0.13 (0.01) ^e^	355.8 (17.8) ^e^	27.8 (3.2)	12.7 (1.5)	36.1 (4.2)
	Total	65.12 (9.16)	–	–	–	70.0 (9.9)	32.2 (5.0)	91.2 (14.3)
Total		–	–	–	–	74.7 (7.0)	34.0 (3.3)	125.4 (9.2)

Different letters indicate significant differences for fine roots within columns (Bonferroni test, p < 0.05).

^a^ n = 44.

^b^ n = 54 or 55.

^c^ Average per tree values calculated from Montagnoli et al. (2021); n = 10.

^d^ Average value for first-order roots used for all root orders; n = 22.

^e^ Average value for all taproot depths; n = 24.

Trees per hectare = 1075.

For fine roots, sampling depth was not significant for C concentration (p = 0.079) but was for N concentration (p <0.001). Fine roots in the forest floor had the highest N concentration, and the 0–10 cm depth had a higher N concentration than the other depths, which were not significantly different (**Table 2**). Depth significantly affected C:N (p <0.001), which increased from the forest floor to the deepest mineral layer (20–30 cm). Intermediate values were observed at 0–10 cm and 10–20 cm (**Table 2**).

For the taproot, depth was not significant for C (p = 0.738; $\bar{\text{x}}$ = 45.9% ± 0.1) and N (p = 0.725; $\bar{\text{x}}$ = 0.13% ± 0.01) concentrations or C:N (p = 0.300; $\bar{\text{x}}$ = 356 ± 18). Similarly, for first-order lateral roots inside the cage, the sampling position was not significant for C (p = 0.621; $\bar{\text{x}}$ = 46.1% ± 0.1) and N (p = 0.643; $\bar{\text{x}}$ = 0.130% ± 0.004) concentrations or C:N (p = 0.360; $\bar{\text{x}}$ = 362 ± 15). The coarse roots held 95% and 73% of all C and N associated with roots on a per hectare basis, with most of that (85% C and 62% N) held in the taproot and first-order lateral roots (**Table 2**).

Using the FVS equations, we estimated total aboveground biomass (TAB) to be 226.5 kg tree^-1^ and root biomass to be about 21.5% of TAB: 48.7 kg tree^-1^ (**Table 1**). Assuming 1075 trees ha^-1^, these equations estimated 52.4 Mg ha^-1^ of root biomass. Our measured wood density values ranged from 410 to 470 kg m^-3^, with a mean of 434 ± 8 kg m^-3^. Maximum root diameter was not significant for, or correlated with, density (p = 0.792, R^2^ = 0.0106). Using this wood density value, we calculated that coarse roots (lateral and tap) contributed 42.2 and 25.8 Mg ha^-1^, respectively (**Table 2**). Fine roots contributed another 4.6 Mg ha^-1^, bringing the total to 74.7 Mg ha^-1^ (**Table 2**). Using the FVS value for C concentration (0.50) and the average concentration values we determined for fine (0.41) and coarse (0.46) roots, FVS models 26.2 Mg C ha^-1^, whereas we measured 34.0 Mg C ha^-1^.

## 4 Discussion

We sampled *P. ponderosa* fine roots in early June, considered within the peak time for their occurrence (Anderson et al., 2008). For length, diameter, and biomass, fine roots in the forest floor had lower values than those in the mineral soil, consistent with other research conducted on *P. ponderosa* in Idaho (Dumm et al., 2008). Given that our forest floor layer was generally thin (~3–4 cm; data not shown) and under a dense canopy in this non-thinned stand, it likely was prone to drier moisture conditions less hospitable to root growth than those occurring in the mineral soil (e.g., Harvey et al., 1986). The greater SRL for fine roots in the forest floor is consistent with an acquisitive strategy (Montagnoli et al., 2019b) toward exploiting the nutritional zone between the forest floor and mineral soil (Harvey et al., 1986). For fine root C concentration, our value of 41% concurs with Page-Dumroese and Jurgensen (2006) but is less than the 54% reported for ~20-year-old stands of outplanted *P. ponderosa* in California (McFarlane et al., 2009). For fine root N concentration, however, our value of 0.84% is similar to the 0.7% reported by McFarlane et al. (2009) but lower than the 1.2% average reported for a variety of field sites and conifer species across the Pacific Northwest, USA (Page-Dumroese and Jurgensen, 2006). This is not surprising given that seasonal fluctuations in fine root growth and differences in the sampling points across studies can affect this value (Zhao et al., 2024).

For coarse roots, our measured C concentration in first-order lateral roots was 46%, similar to that observed for *P. ponderosa* in Arizona (Klemmedson, 1975). In our study, we applied the C and N concentrations measured in first-order lateral and tap roots, to second- and third-order roots. We assumed that because our first-order lateral and tap roots constituted most of the coarse root biomass of our trees (85%; **Table 2**) and because most of that biomass occurs within the root cage (92%; Montagnoli et al., 2021) where we sampled in this study, our estimates of overall coarse root C and N represent of most of the root system.

For *P. ponderosa* wood, Lowery (1984) reports a range-wide wood density of 448.5 kg m^-3^ at 12% moisture content, which is equivalent to 400 kg m^-3^ oven dry (table 4-6a in Glass and Zelinka, (2021)). We calculated a root wood density of 434 kg m^-3^, about 8% more than that of the stem. This is not surprising given that the wood density in roots has been shown to be higher than that of stems in deciduous (e.g., Zhao et al., 2018) and coniferous (e.g., Lemay et al., 2018) species. Our measured wood density seems reasonable for scaling coarse root volumes to biomass.

The base biomass equations (i.e., Jenkins et al., 2003) and FVS assume a root C concentration of 50%, a value higher than our fine- (41%) and coarse-root (46%) C concentrations. Moreover, the base equations modeled root biomass to be about 22 Mg ha^-1^ lower than our calculated values (52 vs. 75 Mg ha^-1^). The net result was that the base equations modeled about 8 Mg ha^-1^ less C than our calculated values of root C (26 vs. 34 Mg ha^-1^). These results are not surprising given that root biomass (and other allometric variables) can vary because of tree age, site quality, tree density, and past silvicultural treatments (e.g., Zhang et al., 2021). Undoubtedly, values can also be influenced by the research objectives and resultant methods employed. Our root architecture questions required meticulous excavation of a small number of trees using an air-spade to ensure sampling roots to about 1 cm in diameter (e.g., Dumroese et al., 2022; Montagnoli et al., 2020), whereas an objective of Zhang et al. (2021) was to examine silvicultural effects; they sampled a greater number of sites and trees with a coarser excavation method involving explosives. As Zhang et al. (2021) conclude, accurate measures of root biomass and C require site-specific measurements. This underscores the need for more root data across a variety of sites toward improving local calibrations and model results—the highlighted priority of Woodall et al. (2025).

In concert with the general need for more root research overall, our *a posteriori* work reported here highlights a need for more *a priori* considerations in data to collect. Our estimates of C and N pools were limited to a single sample of fine roots from a narrow zone around the boles, rather than systematically across the area beneath the stand. We did not measure the fraction of roots with diameters between 2 mm and 1 cm, a root fraction rarely reported in the literature. Our C:N measures relied solely on the first-order and tap root fractions and did not include any contributions of bark (biomass or C:N). And, in the case of this study, a more thorough sampling of the aboveground portion of the trees could have provided a better estimate of overall landscape C and N pools. Because of the inherent difficulty of excavating entire root systems of large trees, our sample size was small. Most of the limitations of our work, including a dedicated analysis that includes roots (1 cm < d < 2 mm) that we missed in the present work, could be mitigated through application of existing technologies, and development of new technologies may provide opportunities to increase sample sizes. For example, recent work suggests that pairing Terrestrial Laser Scanning with Quantitative Structure Models is a viable measurement method, with the added benefit that excavated coarse roots can be measured *in situ* in the forest rather than transported back to laboratories (Montagnoli et al., 2024).

In conclusion, this *a posteriori* study of fine root traits and fine and coarse root C and N concentrations allowed us to estimate landscape C and N pools for a 32-year-old stand of *P. ponderosa* in northern Idaho, USA. Our direct-measured estimates differed from those generated by the FVS model equations, not surprisingly given inherent variation across the 11 million ha where this species grows. Modelers of forest C allocations in ecosystems may find these data useful in refining current models.

## Data Availability

The datasets presented in this study can be found in online repositories. Data are archived and freely available at Montagnoli et al. (2021, 2026).
